# Clinical and economic impacts of optical coherence tomography prior to cataract surgery in a low-to-middle-income country

**DOI:** 10.3389/fopht.2026.1685589

**Published:** 2026-02-02

**Authors:** Mansur Inkarbekov, Mukhit Kulmaganbetov, Galiya Bazarbekova, Almagul Baiyrkhanova, Benjamin Thompson

**Affiliations:** 1 Ophthalmology Department, Kazakh-Russian Medical University, Almaty, Kazakhstan; 2 Department of Epidemiology, Biostatistics and Evidence-Based Medicine, Al-Farabi Kazakh National University, Almaty, Kazakhstan; 3 Department of Glaucoma, Kazakh Eye Research Institute, Almaty, Kazakhstan; 4 Quantum Optics Lab, Centre for Eye and Vision Research (CEVR), Hong Kong, Hong Kong SAR, China; 5 Department of Oncology with Radiology Course, Kazakh-Russian Medical University, Almaty, Kazakhstan; 6 Department of Health Policy and Management, Kazakh National Medical University named after S. D. Asfendiyarov, Almaty, Kazakhstan; 7 Department of Medicine, International Kazakh-Turkish University named after K.A. Yassawi, Turkestan, Kazakhstan; 8 School of Optometry and Vision Science, University of Waterloo, Waterloo, ON, Canada

**Keywords:** cataract, cost-effectiveness, Kazakhstan, optical coherence tomography, visual acuity

## Abstract

**Background:**

Integrating optical coherence tomography (OCT) into the pre-surgical stage of cataract management holds significant clinical and economic potential, particularly in low-to-middle-income countries like Kazakhstan.

**Objective:**

This study aimed to evaluate these impacts by prospectively recruiting 225 patients, aged 71 ± 6 years, from two ophthalmology surgical centers in Almaty, Kazakhstan.

**Methods:**

Patients undergoing phacoemulsification of cataracts with monofocal intraocular lens (IOL) implantation between January 2022 and December 2023 were divided into two groups: the OCT group (n=75) received pre-operative OCT, while the Control group (n=150) did not. Visual acuity (VA) was measured pre- and post-surgery, and the cost-effectiveness of the surgical strategies was analyzed.

**Results:**

The OCT group experienced a significantly greater improvement in VA (-0.647 ± 0.232 logMAR) compared to the Control group (-0.543 ± 0.244 logMAR), with a notable interaction between time and group (F(df) = 396.5, p< 0.001). Additionally, pre-existing concomitant diseases were diagnosed in 24.8% of cases in the OCT group and 31% in the Control group.

**Conclusion:**

The economic analysis revealed that the integration of OCT facilitated combined surgical procedures during a single operative session, thereby reducing overall costs. These findings suggest that implementing OCT in the pre-surgical stage of cataract management can significantly enhance visual outcomes and decrease the cost of ophthalmology services in Kazakhstan.

## 1 Introduction

Cataract, the leading cause of visual impairment globally (1, 2), is characterized by clouding of the crystalline lens, potentially leading to blindness if left untreated (3). Various factors, including ageing, genetics, medical conditions, eye trauma, and ultraviolet radiation exposure, contribute to cataract development (4–6).

Surgical intervention, specifically phacoemulsification (7, 8), is the primary treatment, involving the use of ultrasound to remove the clouded lens and implant an artificial intraocular lens (IOL). Cataract surgery is safe and effective, typically performed on an outpatient basis, with a short recovery period, resulting in improved vision and significant improvement in quality-of-life post-surgery (9–11). Both conventional extracapsular surgery and manual small incision operations are cost-effective alternatives to phacoemulsification (12–15). However, phacoemulsification is considered the standard surgical procedure recommended within the clinical guidelines of the Kazakhstan Ophthalmic Society (16).

Optical coherence tomography (OCT) can enhance the management of cataracts (17). OCT is a non-invasive imaging technique that provides high-resolution cross-sectional images of the eye (18–20), allowing for detailed visualization and assessment of the anterior and posterior ocular structures (21, 22). In cataract management, OCT can aid in preoperative planning by providing precise measurements of the anterior chamber depth, lens thickness, and axial length. These measurements are essential for selecting the appropriate IOL power and calculating the desired refractive outcome (23). Additionally, OCT can identify any coexisting vitreomacular pathologies (24, 25) and enable retinal layer thickness evaluation to detect glaucomatous degeneration of ganglion cell axons (26, 27), which may impact postoperative visual outcomes and can be treated during the surgical appointment made for cataract surgery.

Despite its potential advantages, the initial financial investment required for acquiring OCT equipment may pose a challenge for low- and middle-income countries, including Kazakhstan’s public and private hospitals, especially in remote regions. The patterns of clinical practice in Kazakhstan provided an opportunity to directly compare outcomes and costs of cataract surgery with and without OCT. We hypothesized that pre-operative OCT would lead to a greater treated eye visual acuity improvement and reduce the cost of ophthalmology services by enabling combined surgical procedures during a single operative session. As a major public health concern, cataract is expected to rise in Kazakhstan (28) due to the ageing of Kazakhstan’s population (29) and environmental factors including air and water pollution caused by the growth of transport highways and waste generation (30, 31).

The primary objective of the present study was to evaluate the clinical and economic impact of integrating OCT into the pre-surgical stage of cataract management.

## 2 Materials and methods

### 2.1 Intervention and design

We compared the improvement in visual acuity following cataract surgery in two groups of patients treated by the same ophthalmologist (MI) working in two centers. One group had pre-operative OCT, and the other did not. In addition, we evaluated the cost-effectiveness of two surgical strategies: 1) one combined surgical appointment integrating cataract surgery along with any other necessary eye surgeries (e.g., trabeculectomy for glaucoma, vitrectomy for vitreomacular traction), facilitated by pre-surgical OCT diagnosis. This strategy avoids the need for multiple separate surgical bookings, pre-operative assessments, and anesthesia events, significantly reducing facility, administrative, and patient burden costs. 2) multiple surgical appointments, one for cataract surgery (without pre-surgical OCT) and subsequent appointments for any other necessary surgeries diagnosed later. This strategy incurs the full costs associated with each distinct surgical session. The comparisons of clinical outcomes and costs were made between two ophthalmology surgical centers in Almaty, Kazakhstan.

### 2.2 Participants and group selection strategy

Inclusion criteria were phacoemulsification treatment for cataract with the implantation of a monofocal intraocular lens (IOL) delivered between January 2022 and December 2023. Exclusion criteria were pre-existing ocular pathologies that may affect the outcomes of cataract surgery, previous ocular surgeries or trauma, pre-existing conditions that may confound the assessment of visual acuity improvement, systemic diseases or conditions that may affect ocular health.

A total of 225 patients were prospectively recruited. The sample size was determined based on the availability of eligible patients within the study period and the capacity of the two clinics. The distribution of participants between the two groups was influenced by the clinical practice patterns and the logistical considerations of the surgical centers. The surgeries were performed at two clinics: Private Clinic EyeDoctor and State City Clinic No. 13, both located in Almaty, Kazakhstan. If a patient underwent surgery for both eyes during the study period, only the first eye surgery was included in the analysis.

Group allocation was determined by clinical setting (availability of OCT) and corresponding surgical strategy. The same experienced surgeon (MI) performed all procedures to minimize surgical technique variation. However, the OCT group was recruited from a private clinic and the Control group from a state clinic, introducing potential confounding factors such as differences in patient socioeconomic status, perioperative care protocols, or unmeasured clinical characteristics. The OCT group (n=75) consisted of patients who had pre-operative OCT as they were undergoing a combined surgical appointment for cataract surgery and any other necessary eye surgeries. The Control group (n=150) comprised patients who did not have pre-operative OCT.

### 2.3 Eye examination

Visual acuity (VA) was measured using the uniformly illuminated (100 lux) Sivtsev table, which consists of rows of Cyrillic letters, with each row representing a specific VA level. The viewing distance for VA testing was 6 meters and results were recorded as a logarithm of the minimum angle of resolution (logMAR). VA was measured pre-operatively and 7 days post-surgery. While 7 days represents a standard initial follow-up in our clinical settings, allowing for the early detection of surgical complications, it is insufficient for evaluating stable refractive outcomes. Transient factors like corneal oedema or inflammation may influence early postoperative VA. These measurements, therefore, represent early postoperative outcomes rather than final visual acuity.

Comprehensive eye and vision examinations also included subjective refraction for the assessment of the best corrected VA, intraocular pressure (IOP) measurement, slit lamp biomicroscopy, indirect ophthalmoscopy, ultrasonography, and ocular biometry measurement with the calculation of intraocular lens power using the IOL Master 700 (Zeiss, Germany). Also, medical histories were collected to identify concomitant ocular and systemic diseases. Concomitant eye diseases (glaucoma, vitreomacular traction syndrome and macular hole) were diagnosed pre-surgery for the OCT group, whereas for the Control group, they were detected during the operation and/or post-surgery.

Optical coherence tomography scanning of the posterior eye was conducted for OCT group in the Private Clinic EyeDoctor, whereas the Control group did not receive retinal OCT imaging due to the absence of the device in the State City Clinic No. 13. OCT outcomes used in this study were peripapillary retinal nerve fibre layer (RNFL) thickness and macular neuronal retinal thickness: the region between the inner limiting membrane (ILM) and retinal pigment epithelium (RPE). OCT images were also used for the evaluation of the vitreous and retinal profiles for the detection of vitreomacular traction syndrome (VMTS), epiretinal membrane (ERM) and macular holes (MH).

### 2.4 Statistical analysis

Our primary outcome was VA in the operated eye. Pre- and post-surgical VA was compared with factors of time (pre vs post) and group (OCT vs no OCT) using an analysis of variance (ANOVA). Secondary outcomes included demographic characteristics: between-group age was assessed for normality, and an independent samples t-test was used to compare groups. Gender, treated eye (right or left) and concomitant diseases (glaucoma, vitreomacular traction syndrome, macular hole) were compared using the χ2 test of independence. Inserted IOL power (<18D, 18-19D, 19.1-20D, 20.1-21D, >21.1D) was compared using the Kruskal-Wallis H test for distribution comparison. A significance level of p< 0.05 was set for all tests. The financial cost of pre-OCT and no OCT procedures was calculated using the equation below (1). The economic model was simplified for feasibility in this initial assessment. We did not perform sensitivity analyses on probability coefficients or cost variables, nor did we incorporate indirect costs (e.g., patient travel, productivity loss). While based on actual procedural costs within the Kazakhstani system (16), these limitations should be considered when interpreting results. Data were organized in MS Excel and analyzed using Python.

(1) Cost=∫CS cost×CS probability+CP management cost×CP probability

where CS – cataract surgery and CP – concomitant pathology. The cost calculations (Equations 1, 2) included direct procedural costs (cataract surgery, trabeculectomy, vitreoretinal surgery) and the cost of the OCT scan (US $50). However, the initial capital investment and ongoing maintenance costs for the OCT equipment (amortization) were not incorporated into this model. These amortization costs represent a significant economic consideration, particularly for resource-limited settings, and their exclusion is a limitation of the present cost analysis.

## 3 Results

### 3.1 Demographic characteristics

**Table 1** presents the demographics of the study participants and their baseline clinical characteristics. Pre-existing concomitant diseases were diagnosed before surgery for the OCT group (24.8%), while for the Control group, they were identified during and after cataract surgery (31%). Notably, glaucoma was detected in 20 and 24.4% of cases in the OCT and Control groups, respectively. The rates of vitreomacular traction syndrome and macular hole were 0.8-1.3%, respectively, in both groups. Statistical analysis revealed no significant differences in the prevalence of concomitant diseases between the two groups. Additionally, there were no significant differences in the calculated power of the intraocular lenses between the study groups.

**Table 1:** Demographics and baseline clinical characteristics of study subjects, n=225.

Characteristics	OCT group, n=75	Control group, n=150	p-value
Age			0.077
Mean±SD	70±8	71±6	
Min	58	57	
Max	84	85	
Gender			0.554
Male	34 (45.3%)	68 (45.3%)	
Female	41 (54.7%)	82 (54.7%)	
Treated eye			0.803
Right eye	35 (46.7%)	81 (54%)	
Left eye	40 (53.3%)	69 (46%)	
Concomitant diseases			0.977
Glaucoma	15 (20%)	36 (24.4%)	
Vitreomacular traction syndrome	3 (4%)	8 (5.3%)	
Macular hole	1 (0.8%)	2 (1.3%)	
Intraocular lens power			0.774
**<**18D	29 (38.7%)	52 (34.7%)	
18-19D	8 (10.7%)	19 (12.7%)	
19.1-20D	7 (9.3%)	20 (13.3%)	
20.1-21D	5 (6.7%)	6 (4%)	
>21D	26 (34.7%)	53 (35.3%)	

### 3.2 Clinical outcomes

**Figure 1:**
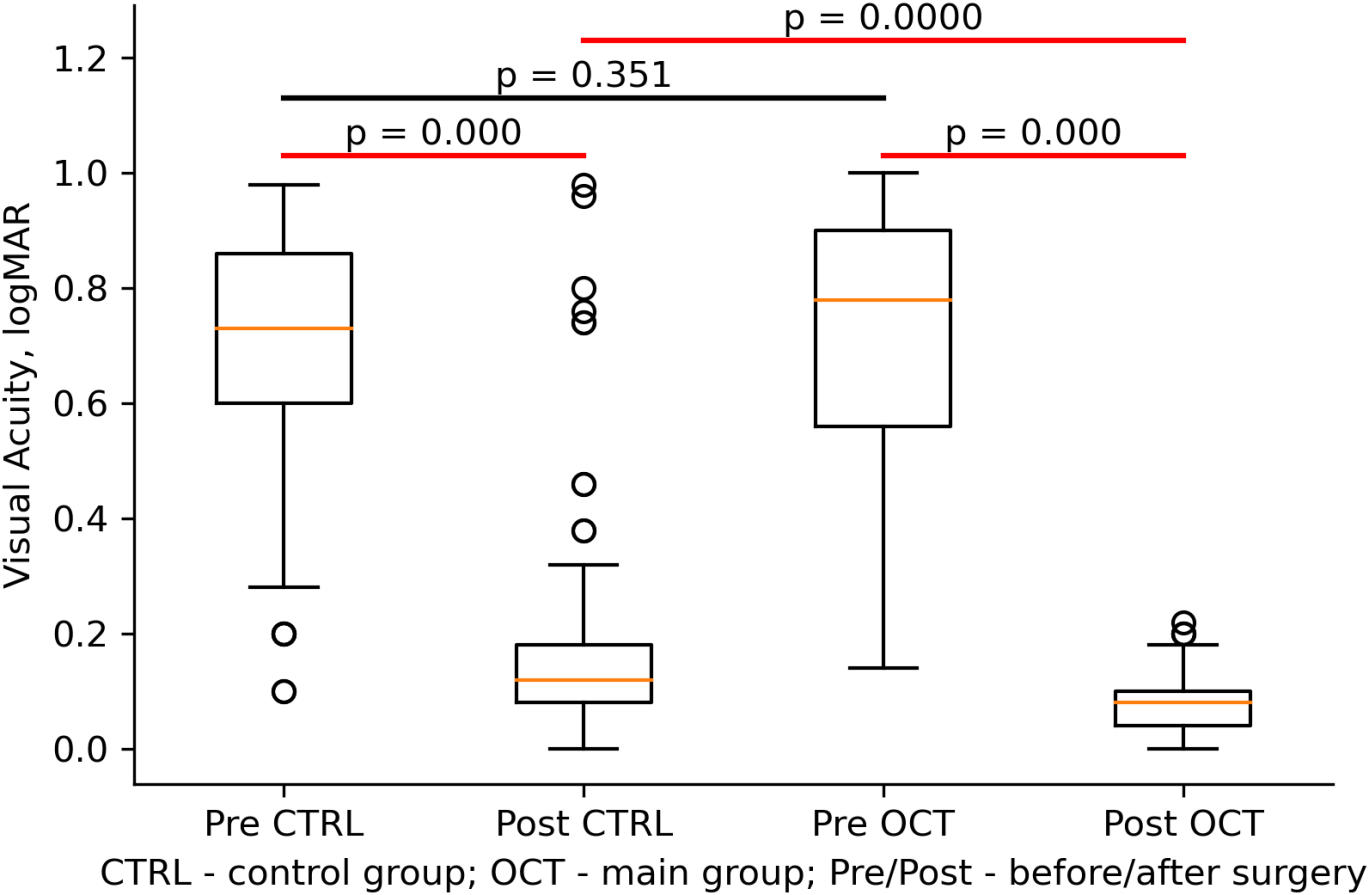
Visual acuity of the Control (CTRL) and OCT groups before (Pre) and after (Post) the cataract surgery. Statistically significant differences were highlighted with the red line, and the orange line shows the mean values and whiskers including lower and upper quartiles.

**Table 2** shows the VA range (logMAR) before and 7 days after the surgery, the number of patients within each range, and the corresponding percentages for each group. The OCT group experienced a significantly greater improvement in VA (-0.647 ± 0.232) compared to the no OCT control group (-0.543 ± 0.244) with a significant interaction between time and group with F(1,223) = 396.5, p< 0.001 (**Figure 1**).

**Table 2:** Visual acuity before and after cataract surgery for the OCT and control groups.

Visual acuity range, logMAR	OCT group, n=75		Control group, n=150	
	Before	After	Before	After
Mean ± SD	0.73 ± 0.233	0.08 ± 0.054	0.70 ± 0.199	0.16 ± 0.013
0.0-0.10	0	55 (73.3%)	2 (1.3%)	63 (42%)
0.12-0.20	2 (2.3%)	19 (25.3%)	4 (2.7%)	61 (40.7%)
0.22-0.30	1 (1.3%)	1 (1.3%)	2 (1.3%)	14 (9.3%)
0.32-0.40	5 (6.7%)	0	6 (4%)	4 (2.7%)
0.42-0.50	4 (5.3%)	0	10 (6.7%)	2 (1.3%)
0.52-0.60	7 (9.3%)	0	16 (10.7%)	0
0.62-0.70	12 (16%)	0	28 (18.7%)	0
0.72-0.80	9 (12%)	0	30 (20%)	2 (1.3%)
0.82-0.90	16 (21.3%)	0	32 (21.3%)	2 (1.3%)
0.92-1.00	19 (25.3%)	0	20 (13.3%)	2 (1.3%)

### 3.3 Cost analysis

**Figure 2:**
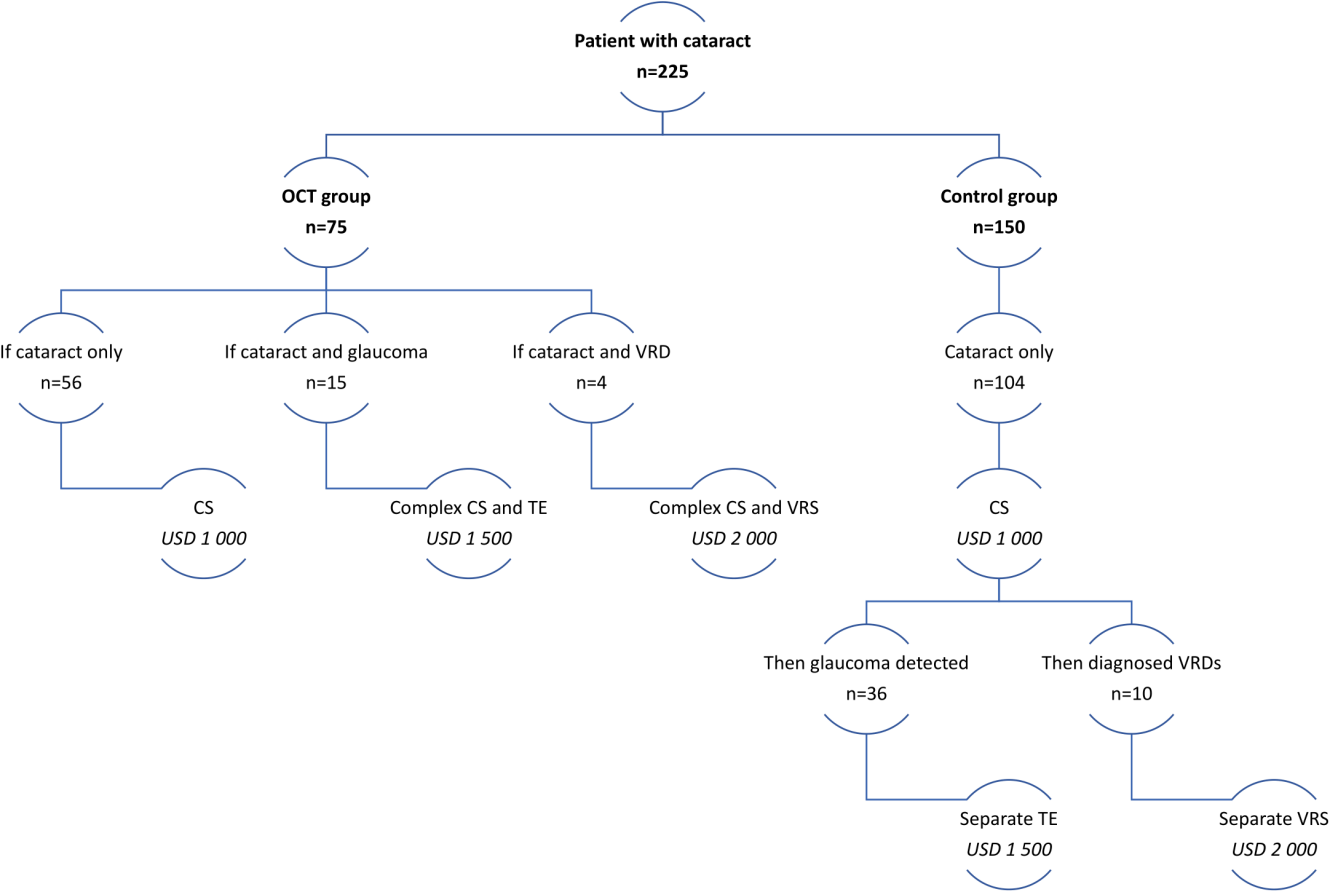
Price comparison for surgical interventions between the OCT and no-OCT control group. CS, cataract surgery; TE, trabeculectomy; VRD, vitreoretinal disease (here: Vitreomacular traction syndrome and Macular hole); VRS, vitreoretinal surgery.

**Figure 2** illustrates the cost of cataract management for each site (OCT vs no-OCT). In the Control group, 24.4% (n = 36) of patients who had glaucoma could have undergone filter surgery (trabeculectomy) at the same surgical visit if their condition had been diagnosed earlier. Additionally, 20% of patients with normal IOP but a thin RNFL and a cup-disc ratio greater than 0.5 (as observed through OCT en-face rendering, color, and red-filter imaging) could have received complex surgery with trabeculectomy. It is worth noting that microinvasive surgery was not included in the clinical protocol for cataract management in Kazakhstan.

Similar considerations apply to patients with vitreomacular traction syndrome. The probability of this syndrome occurring was 4%, with three patients in the OCT group and two patients in the Control group affected. For patients in the OCT group, complex one-time cataract and vitreoretinal surgery was recommended. Conversely, patients in the Control group with vitreomacular traction syndrome were required to wait for separate visits for vitreoretinal surgery to minimize the risk of complications.

To determine the cost of one surgical appointment (separate surgeries) rather than combined surgical procedures during a single operative session, probability coefficients were computed to assess the occurrence of concomitant pathologies for both groups. These coefficients are presented in **Table 3**. The cost calculations (Equation 2) for separate and complex surgeries are presented in **Figure 2**.

**Table 3:** Concomitant pathologies diagnosed before (OCT group, n=75), during and after cataract surgery (Control group, n=150).

Concomitant pathology	OCT group		Control group	
	n	PC	n	PC
Glaucoma	15	0.2	36	0.2
Vitreomacular traction syndrome	3	0.04	8	0.05
Macular hole	1	0.01	2	0.01

PC, probability coefficient.

(2) Cost=∫CP management cost×CP probability+CS cost×0.76

where CP – concomitant pathology, which differs depending on whether it is separate or complex, 0.76 is the probability of Cataract surgery without concomitant pathology.

Despite not factoring into the cost analysis, the management of the OCT group (Equation 3) was found to be more cost-effective than that of the Control group (Equation 4).

(3) $$Cost_{OCT}=\left(1,500\times 0.2\right)+\left(2,000\times 0.04\right)+\left(1,000\times 0.76\right)+50=1,190$$

(4) $$Cost_{ctrl}=\left(2,500\times 0.2\right)+\left(3,000\times 0.05\right)+\left(1,000\times 0.76\right)=1,410$$

Taking into account the aforementioned factors and excluding other potential concomitant pathologies that could be detected using OCT, it can be inferred that the utilization of OCT in cataract management results in lower costs (US $1,190, or KZT 570,336) compared to managing cataracts without this device (US $1,410, or KZT 675,776). The cost values used in Equations 3 and 4 reflect the standard reimbursement rates or patient charges for these specific procedures within the Kazakhstani healthcare system at the time of the study, as established by the Ministry of Health clinical protocols (16) and hospital billing practices. The cost difference between separate appointments and a single combined appointment arises primarily from the elimination of duplicate facility fees, administrative costs, pre-operative assessments, and anesthesia fees associated with scheduling a second, entirely separate surgical session. Performing concomitant pathology surgery during the planned cataract appointment leverages the existing surgical setup, personnel, and anesthesia, leading to significant efficiencies and cost savings for the healthcare system and reduced burden (travel, time off work) for the patient.

## 4 Discussion

This study aimed to evaluate the clinical and economic impact of integrating OCT in the pre-surgical stage of cataract management. While traditional methods like fundus photography and biomicroscopy detect ocular complications such as diabetes-related conditions, full-thickness macular holes, and vein occlusions, OCT offers additional capabilities, identifying epiretinal membranes (32), vitreomacular traction (33), age-related macular degeneration (34), central serous chorioretinopathy (35), retinoschisis (36), and other pathologies.

Within the cohort, we calculated the probability coefficients for pre-existing conditions such as glaucoma (0.2) and vitreomacular traction syndrome (0.04-0.05). Both groups exhibited similar rates of these conditions, indicating no significant differences in the distribution of concomitant diseases, demographics (age and gender), calculated IOL power, or the treated eye (right vs. left). This suggests that these variables did not influence patient allocation and did not confound the comparison between groups.

Using a cost calculation model that factored in the management of separate and complex surgeries and the probability of concomitant pathologies, we found that pre-surgical OCT was more cost-effective than management without it. The OCT group had a lower overall cost (KZT 570,336) compared to the Control group (KZT 675,776), primarily due to early detection and appropriate treatment of concomitant pathologies in a single surgical visit. This indicates better resource allocation and cost savings, particularly relevant for low- and middle-income countries.

Clinically, OCT integration led to significantly greater improvements in visual acuity post-surgery compared to the Control group. Enhanced pre-surgical assessment of vitreoretinal pathologies allowed for more precise surgical planning and better postoperative outcomes, which is crucial for overall quality of life (37, 38). The absolute difference in mean VA improvement between groups was approximately 0.104 logMAR. While statistically significant, the clinical relevance of this difference should be interpreted cautiously. A change of 0.1 logMAR (equivalent to approximately 5 ETDRS letters) is often considered a threshold for minimal clinically important difference (MCID) in visual acuity studies (39, 40). The observed difference, therefore, approaches clinical significance and may represent a meaningful benefit at the population level, though contribution from unmeasured confounders cannot be excluded.

While the current analysis focused on direct procedural costs, a comprehensive evaluation for state hospital implementation must account for capital expenditure, including the purchase of the OCT system, staff training, and maintenance. We estimate that acquiring a standard spectral-domain OCT machine in Kazakhstan requires an investment of approximately US $80,000 to $100,000. Additional operational costs include annual maintenance (≈10–15% of the purchase price) and one-time staff training (≈US $2,000). Assuming a total initial investment of US $100,000, we can project the return on investment based on the savings generated by combined surgical sessions.

Our data indicates that avoiding separate surgeries for concomitant pathologies translates to an average saving of US $220 per patient. Specifically, for every patient undergoing a combined procedure (e.g., cataract + trabeculectomy) instead of two separate surgeries, the healthcare system saves approximately US $880 in facility and administrative fees (calculated by extrapolating the average savings across the 24.8% prevalence of concomitant disease observed in the OCT group). Therefore, approximately 114 combined surgical sessions would be required to offset the full cost of a single OCT machine. Given the observed prevalence of these conditions, a single OCT machine would need to screen approximately 460 patients to generate this number of combined surgeries. In a high-volume state hospital performing 1,500-2,000 cataract surgeries annually, this volume is achievable within 3–6 months. Consequently, despite the upfront capital requirement, long-term projections are highly favorable, with the investment likely being recouped within the first year of operation, followed by substantial cumulative savings over the 5–7 year lifespan of the equipment.

However, this study has limitations. We focused only on vitreomacular traction syndrome and glaucoma, and a larger cohort might reveal different probabilities for other vitreoretinal conditions (40–42). Additionally, we did not study advanced-technology IOL implantations (41), such as multifocal lenses (43), which might affect the variables analyzed. Specifically, multifocal lenses could improve post-op VA at multiple distances compared to monofocal lenses, although they are more expensive and might affect the cost-effectiveness of cataract management. Future research should investigate long-term post-surgical complications like pseudophakic cystoid macular oedema (44) and diabetic retinopathy (45), as they could alter postoperative strategies (46, 47).

First, the non-randomized allocation based on clinic site introduces potential confounding by center-related factors (e.g., patient socioeconomic status, perioperative care protocols) despite the same surgeon and comparable baseline characteristics. Cataract severity was comparable between groups based on preoperative VA (**Table 2**), though other unmeasured complexity factors may exist. Second, the 7-day postoperative VA assessment captures early outcomes but not stable visual acuity; longer follow-up (e.g., 1–3 months) would provide more definitive evidence of visual outcomes. Third, the economic model, while based on real procedural costs within the Kazakhstani system (16), was simplified and did not incorporate amortization costs of OCT equipment, sensitivity analyses, or indirect costs (e.g., patient travel, productivity loss). The probability coefficients for concomitant pathologies were derived from our cohort and may not be generalizable. While the US $50 scan fee partially offsets operational costs, the substantial initial capital investment and ongoing maintenance represent a critical financial barrier, especially for public hospitals or clinics in remote regions of low-to-middle-income countries like Kazakhstan. Future economic models should incorporate amortization costs over the device’s lifespan and patient volume to provide a more comprehensive assessment of long-term cost-effectiveness and return on investment thresholds.

Our findings regarding the cost-effectiveness of OCT align with previous studies in high-income countries. For instance, Chang et al. (2008) reported significant cost savings with OCT compared to fluorescein angiograms, with the equipment investment recovered within four months (48). Balancing financial investment in diagnostic equipment with potential diagnostic errors and missed pathologies could improve cataract management strategies and quality-adjusted life years (49, 50).

Though no device currently provides a realistic post-cataract surgery vision expectation, OCT minimizes ocular complications and treatment expenses (50). Advances in ultrahigh ultrafast OCT systems, adaptive optics (51), multimodal OCT (52), and machine learning algorithms for image analysis (53) enhance OCT’s diagnostic capabilities. Implementing pre-surgical OCT improves cataract patient management by expanding diagnostic options and reducing the risk of underdiagnosing vitreomacular diseases. These outcomes are particularly beneficial for low and middle-income countries like Kazakhstan.

## 5 Conclusion

Despite the challenges associated with the initial investment in OCT equipment (54), especially in remote regions with limited resources, this study demonstrates the significant clinical and economic benefits of OCT in cataract management. Long-term cost savings and improved patient outcomes (55–57) underscore the importance of integrating OCT into pre-surgical protocols. This single-city study provides preliminary evidence suggesting that implementing OCT in the pre-surgical stage of cataract management in settings like Kazakhstan may enhance early visual outcomes and reduce procedural costs by enabling combined surgeries. Further research with multi-center randomized designs, longer follow-up, and comprehensive economic evaluations is warranted to confirm these findings in broader LMIC contexts.

## Data Availability

The raw data supporting the conclusions of this article will be made available by the authors, without undue reservation.

## References

[1] Bourne RR Stevens GA White RA Smith JL Flaxman SR Price H . Causes of vision loss worldwide, 1990-2010: a systematic analysis. Lancet Glob Health. (2013) 1:e339–49. doi: 10.1016/S2214-109X(13)70113-X, PMID: 25104599

[2] Burton MJ Ramke J Marques AP Bourne RRA Congdon N Jones I . The Lancet Global Health Commission on Global Eye Health: vision beyond 2020. Lancet Glob Health. (2021) 9:e489–551. doi: 10.1016/S2214-109X(20)30488-5, PMID: 33607016 PMC7966694

[3] Javitt JC Wang F West SK . Blindness due to cataract: epidemiology and prevention. Annu Rev Public Health. (1996) 17:159–77. doi: 10.1146/annurev.pu.17.050196.001111, PMID: 8724222

[4] Asbell PA Dualan I Mindel J Brocks D Ahmad M Epstein S . Age-related cataract. Lancet. (2005) 365:599–609. doi: 10.1016/S0140-6736(05)17911-2, PMID: 15708105

[5] Robman L Taylor H . External factors in the development of cataract. Eye (Lond). (2005) 19:1074–82. doi: 10.1038/sj.eye.6701964, PMID: 16304587

[6] Shin J Lee H Kim H . Association between exposure to ambient air pollution and age-related cataract: A nationwide population-based retrospective cohort study. Int J Environ Res Public Health. (2020) 17:9231. doi: 10.3390/ijerph17249231, PMID: 33321894 PMC7763970

[7] Kohnen T Baumeister M Kook D Klaproth OK Ohrloff C . Cataract surgery with implantation of an artificial lens. Dtsch Arztebl Int. (2009) 106:695–702. doi: 10.3238/arztebl.2009.0695, PMID: 19946433 PMC2780012

[8] Lapp T Wacker K Heinz C Maier P Eberwein P Reinhard T . Cataract surgery-indications, techniques, and intraocular lens selection. Dtsch Arztebl Int. (2023) 120:377–86. doi: 10.3238/arztebl.m2023.0028, PMID: 36794457 PMC10413970

[9] Yorston D . High-volume surgery in developing countries. Eye (Lond). (2005) 19:1083–9. doi: 10.1038/sj.eye.6701966, PMID: 16304588

[10] Heemraz BS Lee CN Hysi PG Jones CA Hammond CJ Mahroo OA . Changes in quality of life shortly after routine cataract surgery. Can J Ophthalmol. (2016) 51:282–7. doi: 10.1016/j.jcjo.2016.02.004, PMID: 27521668

[11] Han X Zhang J Liu Z Tan X Jin G He M . Real-world visual outcomes of cataract surgery based on population-based studies: a systematic review. Br J Ophthalmol. (2023) 107:1056–65. doi: 10.1136/bjophthalmol-2021-320997, PMID: 35410876 PMC10359559

[12] Asimakis P Coster DJ Lewis DJ . Cost effectiveness of cataract surgery. A comparison of conventional extracapsular surgery and phacoemulsification at Flinders Medical Centre. Aust N Z J Ophthalmol. (1996) 24:319–25. doi: 10.1111/j.1442-9071.1996.tb01602.x, PMID: 8985543

[13] Manaf MR Aljunid SM Annuar FH Leong CK Mansor N . Cost-effectiveness analysis of cataract surgery with intra-ocular lens implantation: extracapsular cataract extraction versus phacoemulsification. Med J Indones. (2007) 16:25. doi: 10.13181/mji.v16i1.252

[14] Jongsareejit A Wiriyaluppa C Kongsap P Phumipan S . Cost-effectiveness analysis of manual small incision cataract surgery (MSICS) and phacoemulsification (PE). J Med Assoc Thai. (2012) 95:212–20., PMID: 22435252

[15] Khan A Amitava AK Rizvi SA Siddiqui Z Kumari N Grover S . Cost-effectiveness analysis should continually assess competing health care options especially in high volume environments like cataract surgery. Indian J Ophthalmol. (2015) 63:496–500. doi: 10.4103/0301-4738.162600, PMID: 26265639 PMC4550981

[16] Clinical protocols of the Ministry of Health of the Republic of Kazakhstan (2017). Available online at: https://diseases.medelement.com/disease/katapakta-2017/15346 (Accessed April 5, 2024).

[17] Nguyen P Chopra V . Applications of optical coherence tomography in cataract surgery. Curr Opin Ophthalmol. (2013) 24:47–52. doi: 10.1097/ICU.0b013e32835aee7b, PMID: 23197267

[18] Fujimoto JG Pitris C Boppart SA Brezinski ME . Optical coherence tomography: an emerging technology for biomedical imaging and optical biopsy. Neoplasia. (2000) 2:9–25. doi: 10.1038/sj.neo.7900071, PMID: 10933065 PMC1531864

[19] Drexler W Morgner U Ghanta RK Kärtner FX Schuman JS Fujimoto JG . Ultrahigh-resolution ophthalmic optical coherence tomography. Nat Med. (2001) 7:502–7. doi: 10.1038/86589, PMID: 11283681 PMC1950821

[20] Kulmaganbetov M Bevan R Want A Anantrasirichai N Achim A Albon J . SVM-Based Optical Detection of Retinal Ganglion Cell Apoptosis. Photonics. (2025) 12:128. doi: 10.3390/photonics12020128 PMC7619084 42147759

[21] Chen S Liu X Wang N Wang X Xiong Q Bo E . Visualizing micro-anatomical structures of the posterior cornea with micro-optical coherence tomography. Sci Rep. (2017) 7:10752. doi: 10.1038/s41598-017-11380-0, PMID: 28883661 PMC5589810

[22] Ang M Baskaran M Werkmeister RM Chua J Schmidl D Aranha Dos Santos V . Anterior segment optical coherence tomography. Prog Retin Eye Res. (2018) 66:132–56. doi: 10.1016/j.preteyeres.2018.04.002, PMID: 29635068

[23] Hipólito-Fernandes D Luís ME Serras-Pereira R Gil P Maduro V Feijão J . Anterior chamber depth, lens thickness and intraocular lens calculation formula accuracy: nine formulas comparison. Br J Ophthalmol. (2022) 106:349–55. doi: 10.1136/bjophthalmol-2020-317822, PMID: 33229347

[24] Wilkins JR Puliafito CA Hee MR Duker JS Reichel E Coker JG . Characterization of epiretinal membranes using optical coherence tomography. Ophthalmology. (1996) 103:2142–51. doi: 10.1016/s0161-6420(96)30377-1, PMID: 9003350

[25] Trichonas G Kaiser PK . Optical coherence tomography imaging of macular oedema. Br J Ophthalmol. (2014) 98:ii24–9. doi: 10.1136/bjophthalmol-2014-305305, PMID: 24934220 PMC4208347

[26] Frenkel S Morgan J Blumenthal E . Histological measurement of retinal nerve fibre layer thickness. Eye. (2005) 19:491–8. doi: 10.1038/sj.eye.6701569, PMID: 15332103

[27] Unterlauft JD Rehak M Böhm MRR Rauscher FG . Analyzing the impact of glaucoma on the macular architecture using spectral-domain optical coherence tomography. PLoS One. (2018) 13:e0209610. doi: 10.1371/journal.pone.0209610, PMID: 30596720 PMC6312265

[28] Kabylbekova A Meirmanov S Aringazina A Orazbekov L Auyezova A . Clinical characteristics of congenital and developmental cataract in Kazakhstan. Indian J Ophthalmol. (2022) 70:4325–30. doi: 10.4103/ijo.IJO_939_22, PMID: 36453339 PMC9940559

[29] Sidorenko AV Eshmanova AK Abikulova AK . Aging of the Population in Kazakhstan. 1. Problems and Opportunities. Adv Gerontol. (2018) 8:12–21. doi: 10.1134/S2079057018010113, PMID: 28968024

[30] Nugumanova L Frey M Yemelina N Yugay S . Environmental problems and policies in Kazakhstan: Air pollution, waste and water. Regensburg: IOS Working Papers (2017). 366.

[31] Alimbaev T Mazhitova ZH Omarova B Kamzayev B Atanakova K . Ecological problems of modern central Kazakh-stan: challenges and possible solutions. E3S Web Conf. (2020) 157:3018. doi: 10.1051/e3sconf/202015703018

[32] Stevenson W Prospero Ponce CM Agarwal DR Gelman R Christoforidis JB . Epiretinal membrane: optical coherence tomography-based diagnosis and classification. Clin Ophthalmol. (2016) 10:527–34. doi: 10.2147/OPTH.S97722, PMID: 27099458 PMC4820189

[33] Cereda M Caimi A Bottoni F Staurenghi G . Optical coherence tomography in eyes with vitreomacular traction. Ophthalmology. (2013) 120:e46–7. doi: 10.1016/j.ophtha.2013.02.032, PMID: 23823522

[34] Alizadeh Y Akbari M Moghadam RS Medghalchi A Dourandeesh M Bromandpoor F . Macular optical coherence tomography before cataract surgery. J Curr Ophthalmol. (2021) 33:317–22. doi: 10.4103/joco.joco_240_20, PMID: 34765821 PMC8579804

[35] Iida T Hagimura N Sato T Kishi S . Evaluation of central serous chorioretinopathy with optical coherence tomography. Am J Ophthalmol. (2000) 129:16–20. doi: 10.1016/s0002-9394(99)00272-x, PMID: 10653407

[36] Ip M Garza-Karren C Duker JS Reichel E Swartz JC Amirikia A . Differentiation of degenerative retinoschisis from retinal detachment using optical coherence tomography. Ophthalmology. (1999) 106:600–5. doi: 10.1016/S0161-6420(99)90123-9, PMID: 10080221

[37] Chan CW Wong JC Chan KS Wong WK Tam KC Chau PS . Evaluation of quality of life in patients with cataract in Hong Kong. J Cataract Refract Surg. (2003) l29:1753–60. doi: 10.1016/s0886-3350(03)00042-7, PMID: 14522296

[38] Keane PA Sadda SR . Predicting visual outcomes for macular disease using optical coherence tomography. Saudi J Ophthalmol. (2011) 25:145–58. doi: 10.1016/j.sjopt.2011.01.003, PMID: 23960916 PMC3729388

[39] Navascues-Cornago M Guthrie S Morgan PB Woods J . Determination of the minimal clinically important difference (MCID) for ocular subjective responses. Trans Vis Sci Tech. (2024) 13:28. doi: 10.1167/tvst.13.8.28, PMID: 39150716 PMC11343006

[40] Huang X Zhang Z Wang J Meng X Chen T Wu Z . Macular assessment of preoperative optical coherence tomography in ageing Chinese undergoing routine cataract surgery. Sci Rep. (2018) 8:5103. doi: 10.1038/s41598-018-22807-7, PMID: 29572456 PMC5865193

[41] Klein BR Brown EN Casden RS . Preoperative macular spectral-domain optical coherence tomography in patients considering advanced-technology intraocular lenses for cataract surgery. J Cataract Refract Surg. (2016) 42:537–41. doi: 10.1016/j.jcrs.2016.01.036, PMID: 27113875

[42] McKeague M Sharma P Ho AC . Evaluation of the macula prior to cataract surgery. Curr Opin Ophthalmol. (2018) 29:4–8. doi: 10.1097/ICU.0000000000000432, PMID: 28902720

[43] Hinnig RB Martins LFS Penha FM . Spectral domain oct for screening of macular diseases prior to multifocal intraocular lens implantation. Int J Retina Vitreous. (2022) 8:77. doi: 10.1186/s40942-022-00427-8, PMID: 36273199 PMC9587646

[44] Henderson BA Kim JY Ament CS Ferrufino-Ponce ZK Grabowska A Cremers SL . Clinical pseudophakic cystoid macular edema. Risk factors for development and duration after treatment. J Cataract Refract Surg. (2007) 33:1550–8. doi: 10.1016/j.jcrs.2007.05.013, PMID: 17720069

[45] Hong T Mitchell P de Loryn T RochtChina E Cugati S Wang JJ . Development and progression of diabetic retinopathy 12 months after phacoemulsification cataract surgery. Ophthalmology. (2009) 116:1510–4. doi: 10.1016/j.ophtha.2009.03.003, PMID: 19501407

[46] Curković T Vukojević N Bućan K . Treatment of pseudophakic cystoid macular oedema. Coll Antropol. (2005) 29:103–5. 16193688

[47] Ghasemi Falavarjani K Parvaresh MM Modarres M Hashemi M Samiy N . Intravitreal bevacizumab for pseudo-phakic cystoid macular edema; a systematic review. J Ophthalmic Vis Res. (2012) 7:235–9., PMID: 23264866 PMC3520593

[48] Chang DL Subramanian ML Legutko PA Daly MK . Cost-Benefit Analysis on the Use of Optical Coherence Tomography versus Fluorescein Angiogram in the Diagnosis of Macular Diseases at the VA Boston Healthcare System, Jamaica Plain Campus. Invest Ophthalmol Vis Sci. (2008) 49:1860.

[49] Leung EH Gibbons A Koch DD . Cost-effectiveness of preoperative OCT in cataract evaluation for multifocal intraocular lens. Ophthalmology. (2020) 127:859–65. doi: 10.1016/j.ophtha.2020.01.049, PMID: 32173111 PMC7311225

[50] Goldhardt R Rosen BS . Optical coherence tomography: critical tool to manage expectations after cataract extraction. Curr Ophthalmol Rep. (2020) 8:129–35. doi: 10.1007/s40135-020-00243-z, PMID: 33094032 PMC7574664

[51] Pircher M Zawadzki RJ . Review of adaptive optics OCT (AO-OCT): principles and applications for retinal imaging [Invited. BioMed Opt Express. (2017) 8:2536–62. doi: 10.1364/BOE.8.002536, PMID: 28663890 PMC5480497

[52] Shirazi MF Andilla J Lefaudeux N Valdes C Schwarzhans F Durand M . Multi-modal and multi-scale clinical retinal imaging system with pupil and retinal tracking. Sci Rep. (2022) 12:9577. doi: 10.1038/s41598-022-13631-1, PMID: 35688890 PMC9187716

[53] Kulmaganbetov M Morgan JE . Application of texture analysis in retinal OCT imaging. In: El-Baz A , editor. Handbook of Texture Analysis. CRC Press, Boca Raton, FL (2024). p. 154–79.

[54] Swanson EA Fujimoto JG . The ecosystem that powered the translation of OCT from fundamental research to clinical and commercial impact [Invited. BioMed Opt Express. (2017) 8:1638–64. doi: 10.1364/BOE.8.001638, PMID: 28663854 PMC5480569

[55] Jindal A Ctori I Fidalgo B Dabasia P Balaskas K Lawrenson JG . Impact of optical coherence tomography on diagnostic decision-making by UK community optometrists: a clinical vignette study. Ophthalmic Physiol Opt. (2019) 39:205–15. doi: 10.1111/opo.12613, PMID: 30994199 PMC6849707

[56] Olson J Sharp P Goatman K Prescott G Scotland G Fleming A . Improving the economic value of photographic screening for optical coherence tomography-detectable macular oedema: a prospective, multicentre, UK study. Health Technol Assess. (2013) 17:1–142. doi: 10.3310/hta17510, PMID: 24225334 PMC4781285

[57] Miller KL Walt JG Mink DR Satram-Hoang S Wilson SE Perry HD . Minimal clinically important difference for the ocular surface disease index. Arch Ophthalmol. (2010) 128:94–101. doi: 10.1001/archophthalmol.2009.356, PMID: 20065224

